# A Comprehensive Review of Radiation-Induced Hydrogels: Synthesis, Properties, and Multidimensional Applications

**DOI:** 10.3390/gels10060381

**Published:** 2024-06-02

**Authors:** Md. Shahriar Ahmed, Mobinul Islam, Md. Kamrul Hasan, Kyung-Wan Nam

**Affiliations:** 1 Department of Energy and Materials Engineering, Dongguk University, Seoul 04620, Republic of Korea; shahriar.che.sust@gmail.com (M.S.A.); knam@dongguk.edu (K.-W.N.); 2 Department of Advanced Battery Convergence Engineering, Dongguk University, Seoul 04620, Republic of Korea

**Keywords:** hydrogel, electron beam irradiation, gamma radiation crosslinking, biomedical engineering, drug delivery, sustainable agriculture

## Abstract

At the forefront of advanced material technology, radiation-induced hydrogels present a promising avenue for innovation across various sectors, utilizing gamma radiation, electron beam radiation, and UV radiation. Through the unique synthesis process involving radiation exposure, these hydrogels exhibit exceptional properties that make them highly versatile and valuable for a multitude of applications. This paper focuses on the intricacies of the synthesis methods employed in creating these radiation-induced hydrogels, shedding light on their structural characteristics and functional benefits. In particular, the paper analyzes the diverse utility of these hydrogels in biomedicine and agriculture, showcasing their potential for applications such as targeted drug delivery, injury recovery, and even environmental engineering solutions. By analyzing current research trends and highlighting potential future directions, this review aims to underscore the transformative impact that radiation-induced hydrogels could have on various industries and the advancement of biomedical and agricultural practices.

## 1. Introduction

Hydrogels are acclaimed for their 3D polymeric water-insoluble networks that possess a notable capacity to absorb substantial amounts of water in relation to their weight, rendering them highly sought-after materials in diverse sectors such as medical, pharmaceutical, food, and agricultural sectors [1,2]. In the medical and pharmaceutical realms, the pivotal role hydrogels play as supportive structures, offering mechanical protection for tissues where cells are either suspended within or attached to the polymeric material, is paramount [3,4]. Equally notable is the utilization of hydrogels in the food industry, where they come into play for encapsulating a myriad of active ingredients, exemplifying their versatility and applicability [5]. Gamma and electron beam radiation techniques stand out as prevalent techniques for crosslinking, compatibilizing, and grafting within various polymer blends and composite systems. Notably, gamma radiation-induced grafting and crosslinking have proven to be effective methodologies for enhancing the properties of polymeric materials across an array of high-performance applications. Gamma rays, a form of high-energy electromagnetic radiation, are used to synthesize and modify hydrogels by crosslinking polymer solutions. In biomedicine, these hydrogels are ideal for controlled drug delivery and wound dressings due to their biocompatibility and moisture retention properties [6]. They can be engineered for specific release rates, allowing extended medication administration and enhanced wound healing. In agriculture, gamma ray-induced hydrogels improve soil water retention and crop yields, particularly in arid regions, by absorbing and gradually releasing water [7]. They can also be combined with fertilizers and pesticides for controlled release, increasing efficiency and reducing environmental impact. Environmentally, these hydrogels aid in wastewater treatment by absorbing pollutants like heavy metals and organic compounds, contributing to water purification and environmental cleanup. Their high absorbency and swelling capacity make them ideal for capturing contaminants from industrial effluents and sewage [8]. Industrially, the responsiveness of these hydrogels to stimuli like pH and temperature enables the development of smart materials and sensors that monitor environmental conditions. Their adaptability supports innovative applications in controlled release systems, responsive textiles, and robotics. Gamma ray crosslinking is known for its efficiency in hydrogel synthesis, offering deep penetration and precise control over the crosslinking process. For instance, gamma ray-crosslinked alginate hydrogels have shown promise in wound healing applications, exhibiting enhanced mechanical strength and biocompatibility [9]. Additionally, agarose hydrogels crosslinked using gamma radiation have demonstrated excellent thermal stability and controlled drug release properties, making them suitable for drug delivery platforms [10]. Furthermore, gamma ray-crosslinked polyacrylamide hydrogels have been utilized for the development of biosensors due to their responsive behavior to environmental stimuli and high sensitivity [11]. These examples illustrate the versatility and efficacy of gamma ray crosslinking in tailoring hydrogel properties for various biomedical applications. Similarly, electron beam radiation crosslinking offers economic advantages over conventional chemical techniques for property enhancement. The discourse turns towards the intricate realm of developing polymeric multi-component systems through controlled high-energy radiation crosslinking, emphasizing the significance of this modulation. Several investigative efforts have focused on the modification of polymeric systems employing controlled doses of gamma radiation, with an emphasis on the radiation-induced grafting of diverse monomers onto the polymer backbone—a pivotal aspect of this transformative process. The scrutiny extends to comparative studies delving into the differing effects of gamma and electron beam radiation on property development, underscoring the versatility and applicability of high-energy radiation-modified polymers across diverse sectors ranging from automotive and insulation to sterilization and biomedical spheres, among others. Gamma radiation serves as a versatile tool that is particularly adept at functionalizing surfaces with stimuli-responsive polymers through the creation of active sites on the polymeric backbone via high-energy radiation exposure. This facilitates intricate reactions between monomers/polymers and active sites, culminating in the formation of side chain grafts. The amendation of polymers using high-energy irradiation through methods like direct or simultaneous techniques finds prominence in this realm, emphasizing the synthesis of smart polymers and coatings leveraging gamma radiation, with a keen eye on applications within the biomedical domain. Shifting focus towards polymer hydrogel networks, characterized by their low crosslinking levels, these intricate structures emerge through the employment of chemical or physical crosslinking techniques, leading to the formation of crosslinking points that can either be covalent or noncovalent. Renowned for their exceptional ability to swell or contract, retaining significant water volumes while maintaining insolubility, hydrogels showcase versatility and adaptability [12,13]. Their malleability allows for natural shaping and notable flexibility under varying pressures—key features that underscore their practicality and utility. These specialized polymer materials, acknowledged for their attributes like water absorption, retention, controlled release capabilities, and diverse functional properties, have garnered substantial attention in recent years owing to the rapid strides in their development. Highlighting their versatility, hydrogels often exhibit desirable characteristics such as responsiveness to stimuli, biocompatibility, reversible physicochemical traits, and more [14]. With a composition and structure reminiscent of human soft tissues, hydrogels find extensive applications in a multitude of sectors, including drug delivery, cell culture, tissue engineering, and various biomedical and biomimetic uses [15,16,17,18]. Moreover, the tremendous potential of functionalized hydrogels in domains like intelligent sensing and environmental remediation underscores their versatility and burgeoning prospects [19]. Noteworthy among the traditional repertoire of polymer hydrogel materials is polyvinyl alcohol (PVA), which has witnessed a resurgence in contemporary research avenues. This water-soluble polymer, stemming from the alcoholysis, hydrolysis, or ammonolysis of polyvinyl acetate (PVAc), gives rise to hydrogels with intricate three-dimensional network structures facilitated through crosslinking and swelling procedures. The allure of PVA-based hydrogels lies in their low toxicity, elevated water absorption capacity, robust mechanical properties including a high elastic modulus and strength, and commendable biocompatibility [20,21,22]. Seizing upon these advantageous attributes, applications of these hydrogels extend across a diverse array of domains including the food industry, forestry applications, and super absorbents, with a pronounced emphasis on the biomedicine realm where they serve pivotal roles in drug delivery mechanisms, tissue engineering frameworks, the development of implanted artificial muscles and organs, biosensors, wound dressings, and soft robotics, showcasing their multidisciplinary relevance and profound impact on various fields. Hydrogels stand out as a significant class of functional materials distinguished by their unique structure, customizable functionalities, and notable properties such as high-water content, interconnected porosity, softness, and flexibility. These characteristics evoke a semblance to biological materials like mucus or the extracellular matrix enveloping cells, tissues, organs, or entire organisms [23,24]. The classification of hydrogels into physical, chemical, or permanent gels depends on the type of crosslinking points they feature [25]. Physical gels are characterized by molecular entanglements and secondary forces, rendering their crosslinks reversible, thus enabling dissolution when exposed to different environmental conditions or when in contact with water for a long time. In contrast, permanent or chemical gels are characterized by networks that have covalent bonds serving as crosslinking locations. Hydrogel creation entails the polymerization and concurrent crosslinking of hydrophilic monomers using polyfunctional crosslinking agents, or by directly crosslinking hydrophilic polymers. However, traces of monomers, initiators, catalysts, and their byproducts may inadvertently introduce undesirable traits such as color, chemical reactivity, or potential toxicity. Consequently, a call for simpler and safer synthetic methods is growing, with single component-based processes gaining traction. Utilizing gamma rays or accelerated electron beams has proven effective in creating hydrogels from water-soluble, biocompatible synthetic polymers like polyacrylic acid, polyvinyl alcohol, polyvinylpyrrolidone, polyethylene glycol, and polyacrylamide [26], polysaccharides [27,28], and polyaminoacids [29,30]. The distinct advantage of employing high-energy irradiation lies in achieving sterilization concurrently with appropriate dosages. Alternatively, other methods that do not require reagents, such as the UV irradiation of polymeric systems that can be directly photo-crosslinked, autoclaving, or thermal treatments using microwave radiation, provide further options. It is essential for a polymer to contain photoactive groups such as cinnamic acid, coumarin, anthracene, and dimethylmaleimide in order to undergo direct photo-crosslinking [31]. On the other hand, polymer hydrogels are synthesized by crosslinking hydrophilic polymers either physically or chemically, with the extent of crosslinking significantly impacting the mechanical and chemical properties of the hydrogel. Various methods are employed to create chemical crosslinks, including photo-polymerization, radical-induced crosslinking, and click chemistry techniques like copper-catalyzed alkyne azide coupling and Michael additions [32]. Fine-tuning the crosslink density allows for the modulation of (bio)molecule release, hydrogel rigidity, cellular signaling, and the internal water volume. Control over the crosslink density can be achieved through degradative or constructive molecular events, triggered by factors like (UV) light, pH, enzymatic activity, or reactive oxygen species (ROS). The release of (bio)molecules from the hydrogel matrix is typically accomplished through degradation processes such as triggered crosslinker cleavage, whereas constructive molecular processes like secondary radical-mediated crosslinking and sequential photoinduced crosslinking enhance crosslink density [33]. Enhancing the crosslink density is often pursued to improve mechanical properties such as rigidity, yield stress, or healing capabilities. To achieve the macroscopic contraction of dextran hydrogels, a *γ*-radiation-triggered secondary crosslink strategy is introduced that allows direct control over the density of secondary crosslinks post-initial hydrogel formation. This innovative approach underscores the translation of molecular events like secondary crosslinking into macroscopic movement, exemplified through hydrogel contraction. Leveraging *γ*-radiation enables the generation of radicals on unsaturated polymer chains, facilitating crosslink formation and finding widespread application [34]. The efficiency of crosslinking via gamma radiation lies in its capacity to forgo monomers, initiators, or catalysts which may pose risks in biological applications. By adjusting the radiation dose, control over the crosslink density can be attained, offering versatility in managing properties like rigidity [35]. Extended irradiation leads to continued crosslink formation, resulting in material contraction. This phenomenon, as observed by Angelini et al., demonstrates material contraction in 3% gelatin solutions under a *γ*-irradiation dose surpassing 50 kGy [36], showcasing the high sensitivity and effectiveness of gamma radiation-induced crosslinking.

The main goal of this review is to elucidate the development, characteristics, and multifaceted applications of radiation-induced hydrogels, emphasizing their synthesis via gamma, electron beam, and UV radiation methods. Radiation-induced hydrogels have emerged as pivotal materials in the realm of biomedical engineering, notably in wound healing, due to their unique properties such as high-water content, biocompatibility, and tailored degradability. Furthermore, their utility extends into agriculture, where they contribute to water retention and the controlled release of agrochemicals, enhancing crop productivity in challenging environmental conditions. This paper seeks to compile and analyze recent advancements in the synthesis of these hydrogels, comparing the efficiency and outcomes of different radiation techniques. Moreover, we aim to explore the innovative integration of these hydrogels in biomedical applications, particularly in drug delivery systems and tissue engineering, as well as their rising significance in agricultural practices. By providing a comprehensive overview of current research and potential future directions, this review intends to highlight the transformative potential of radiation-induced hydrogels in science and technology, fostering a deeper understanding and expanded utilization in various disciplinary contexts.

## 2. Synthesis and Properties of Hydrogels

### 2.1. Impact of Gamma and Electron Beam Radiation

Scientists have been studying the impact of high-energy radiation, such as gamma and electron beam radiation, on polymers for over thirty years. This research aims to understand how radiation can be used to achieve the crosslinking, grafting, and compatibilization of polymers [37]. The significance of radiation-exposed polymeric molecules in diverse global applications is widely acknowledged [38]. Over the past thirty years, different constituents from radiation-treated polymers, including customized polymers, polymer mixtures, and hybrids, have been extensively employed in automotive, construction, aerospace, nuclear, defense, electrical, and electronic applications which require high temperatures. Gamma and electron beam radiation play a pivotal role in transforming industrial polymers such as LDPE, HDPE, Nylon-6, Nylon-6 6, EPDM, POE, silicone elastomer, EVA copolymer, and others [39].

The ionization introduced by gamma radiation in polymeric chains triggers chain crosslinking and scission through a mechanism involving free radicals, with the extent of the crosslinking being contingent on factors like polymer composition, phase structure, radiation dosage and duration, and characteristics of the radiation source. The exposure of polymers to gamma radiation has emerged as a prevailing method for altering polymer structure, instigating polymerization, facilitating grafting, sterilization, and fostering the crosslinking of various thermoplastics and elastomers. Innovations arising from gamma radiation processes are highly prized for their suitability in high-performance applications; explorations in polymer radiation technology have unlocked avenues for superior performance in applications of great commercial relevance in packaging, automotive, and electronics sectors [40]. Gamma radiation-induced crosslinking and surface adjustments have elevated the mechanical, thermal, chemical, electrical insulation, and environmental traits of polymers, rendering them apt for demanding applications prevalent in space exploration, automotive industries, construction, nuclear facilities, and defense applications [41,42].

### 2.2. Gamma Radiation-Induced Hydrogel Synthesis

Hydrogel fabrication using gamma radiation involves applying high-energy gamma rays to induce crosslinking within hydrophilic polymer networks. This method utilizes isotopes like cobalt-60 to uniformly crosslink the polymer chains, transforming the precursor solution into a stable, three-dimensional hydrogel. This technique enhances the mechanical strength and stability of hydrogels without requiring chemical crosslinking agents. Hydrogel systems crafted through gamma irradiation have gained considerable attention in recent years. When an aqueous polymer solution is irradiated, radicals form on polymer chains, and water molecules undergo radiolysis, producing hydroxyl radicals that also interact with polymer chains, resulting in macro-radical formation. These macro-radicals then recombine across different chains, leading to covalent bond formation and ultimately, a crosslinked structure [43,44]. The utilization of gamma ray irradiation for living free-radical polymerization offers several benefits for practical applications. The advantages are that it can be controlled easily, it is environmentally friendly, and it can be used to create and sterilize the hydrogel in the same step, all while maintaining a room temperature and allowing for high penetration rates [45,46]. The precision of gamma radiation allows hydrogels to be engineered with specific release rates, enabling extended medication administration and enhanced wound healing. These hydrogels can be loaded with growth factors and other therapeutic agents, providing a moist environment conducive to tissue regeneration, and reducing infection risk [22]. In agriculture, gamma ray-induced hydrogels improve soil water retention and crop yields, especially in drought-prone areas, by absorbing and gradually releasing water. They can also combine with fertilizers and pesticides for controlled release, increasing efficiency and reducing environmental impact [47].

Environmentally, these hydrogels aid in wastewater treatment by absorbing pollutants like heavy metals and organic compounds, contributing to water purification and environmental cleanup. Their high absorbency and swelling capacity make them ideal for capturing contaminants from industrial effluents and sewage [48]. Industrially, their responsiveness to stimuli such as pH and temperature enables the development of smart materials and sensors. These hydrogels can be used in devices that monitor environmental conditions, providing real-time data for industrial processes and safety monitoring. Overall, gamma ray-induced hydrogels offer versatile and valuable solutions across multiple fields, driven by their unique properties and the precise interaction between gamma rays and polymers. Importantly, the radiation technique is environmentally friendly since there is no need for extra chemicals that would introduce harmful contaminants into the networks of polymers, such as chemical initiators and crosslinkers. This method is particularly advantageous for biomedical applications, where even minimal contamination is undesirable. Moreover, gamma ray irradiation is commonly utilized to sterilize biomedical devices for veterinary and medical purposes [49,50]. Hydrogels were developed by exposing them to gamma ray radiation at different doses (26, 64, 96, and 124 kGy) in the presence of air at room temperature. This was performed using a Gamma Cell 220 type 60Co g irradiator, with a constant dose rate of 0.40 kGy h1. The hydrogels were made using poly(N-vinyl-2-pyrrolidone) (PVP) and K_2_S_2_O_8_, with different levels of crosslinking densities and molecular weights [51].

**Figure 1:**
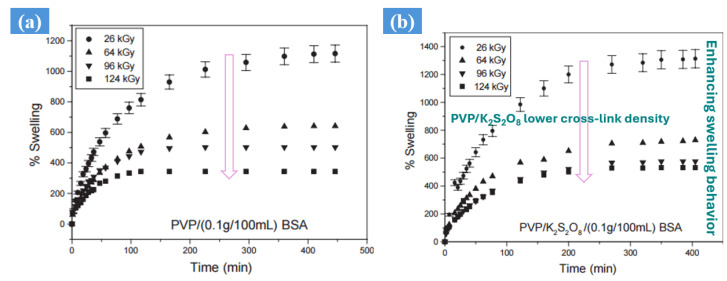
(**a**) Swelling percentages of PVP gels in 0.1 g/100 mL BSA solution across various radiation doses and (**b**) swelling percentages of PVP/K_2_S_2_O_8_ gels in 0.1 g/100 mL BSA at different radiation doses, reproduced with permission from ref. [51]. Copyright, Elsevier.

Scientists studied how these hydrogels behave when they come into contact with a Bovine Serum Albumin solution. They looked at how much the hydrogels swell and spread out, focusing on how different radiation doses affect them (as shown in Figure 1). They found that higher radiation doses increased the crosslinking in the hydrogels, making them swell differently. This research shows how important radiation is in changing the structure and swelling properties of hydrogels, providing useful information about how these materials evolve and behave.

**Figure 2:**
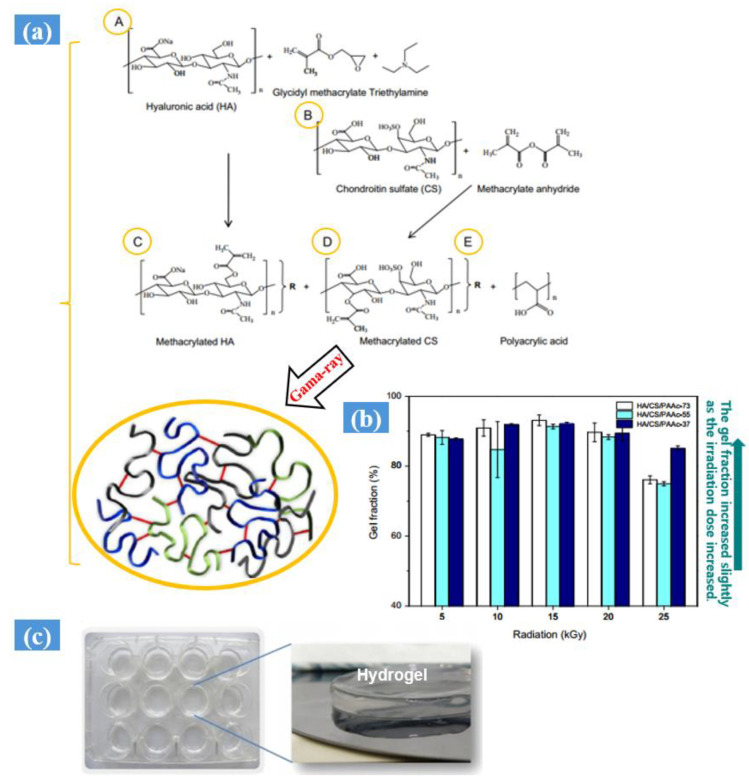
(**a**) Synthetic diagrams for: (A) HA, (B) CS, (C) methacrylated HA (MA-HA), (D) methacrylated CS (MA-CS), and (E) PAAc. (**b**) HA/CS/PAAc-55 hydrogel produced via gamma irradiation. (**c**) Influence of irradiation dose on gel fraction (%) in HA/CS/PAAc hydrogels. Images are reproduced with permission from ref. [52]. Copyright, Elsevier.

In order to achieve a biocompatible matrix suitable for skin tissue engineering, gamma ray irradiation was used to create HA/chondroitin sulfate/polyacrylic acid hydrogel systems without needing additional initiating or crosslinking agents [52] (Table 1). The production involved gamma ray exposure to facilitate free-radical copolymerization and the crosslinking of the glycosaminoglycans HA, CS, and the synthetic ionic polymer PAAc (as presented in Figure 2). The gelation rate of these HA/CS/PAAc hydrogels demonstrated an increase with irradiation doses up to 15 kGy, achieving gel fractions between 91 and 93% at 15 kGy [53,54]. This result corroborates the fact that PAAc’s three-dimensional network formation and gel fraction enhancement are direct outcomes of increased irradiation exposure, substantiating the effectiveness of this method in developing hydrogel systems targeting tissue engineering objectives using FE-SEM. It was observed that HA/CS/PAAc hydrogels had highly porous cross-sectional structures. By crosslinking a linear polymer with radiation, a three-dimensional polymeric network is created that is capable of adsorbing water without dissolving.

A different group of researchers discovered that starch/(EG-co-MAA) polymeric hydrogels were synthesized through gamma-induced radiation copolymerization, where methacrylic acid (MAA) and ethylene glycol (EG) were grafted onto starch. The composition of these hydrogels, including the gel content, was observed to vary with several factors such as the proportion of starch used, the EG:MAA ratio, the irradiation dose, and the crosslinking density. A number of parameters were examined to determine how much swelling these hydrogels could produce, including starch content, EG:MAA composition, irradiation dose, immersion liquid type, pH, and ambient temperature. It was found that the starch/(EG-co-MAA) hydrogels achieved equilibrium swelling in water within 72 h, demonstrating the hydrogels’ responsiveness to environmental conditions and their potential for various applications [60].

**Figure 3:**
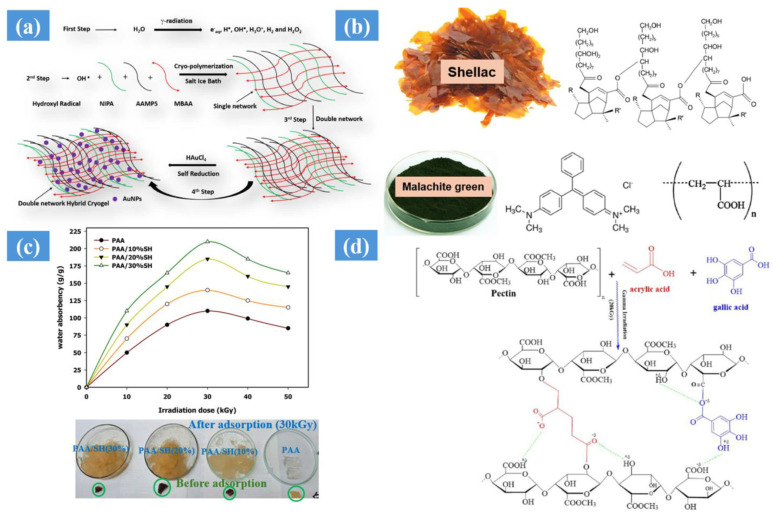
(**a**) Cryo-polymerization mechanisms for single and double network pure and hybrid cryogels via *γ*-radiation [61]. (**b**) Structures of shellac and malachite green. (**c**) Impact of irradiation dose on water absorbency for polyacrylic acid and polyacrylic acid/(10, 20, and 30%) shellac [64]. (**d**) Proposed reaction pathway for the synthesis of PC-PAAc/GA hydrogel [65].

In a pioneering study conducted in 2023, the development of acrylamide-methyl-propane sulfonic acid (AAMPS)-based hydrophilic cryogels through gamma radiation at a low pH level was explored (as shown in Figure 3a). This work included the production of gold hybrid cryogels via a self-reduction method under ambient conditions [61]. Demonstrating a high efficiency in degrading Congo red dye with NaBH_4_, these cryogels reveal significant potential for environmental remediation. Smart hydrogels and cryogels, recognized for their broad utility in sectors such as drug delivery, catalysis, and sensor technology [62,63], benefit from gamma radiation’s deep penetration and scalability. This research highlights a methodological advantage over UV radiation techniques due to gamma radiation’s ability to produce cryogels of any thickness, overcoming the limitations of UV methods in penetrating larger samples. The simplified production process avoids the pH adjustments necessary for AAMPS, presenting gamma radiation as a versatile and efficient approach for cryogel synthesis. The documentation of the cryo-polymerization mechanism in Figure 3a further illustrates the process’s adaptability and efficiency. This study not only advances the synthesis of hydrophilic cryogels but also emphasizes the significance of gamma radiation in creating scalable and versatile cryogel systems.

Another study conducted by a different research group delved into the development of a novel superabsorbent hydrogel comprising polyacrylic acid and shellac. The process involved utilizing gamma irradiation for the purpose of adsorbing and removing malachite green dye. The primary aim was to fabricate a highly absorbent hydrogel by combining polyacrylic acid with environmentally friendly shellac to effectively eliminate malachite green dye from aqueous solutions. The study focused on investigating the adsorption of malachite green dyes through the utilization of polyacrylic acid/shellac hydrogels. These hydrogels were synthesized by blending aqueous solutions of polyacrylic acid and shellac at various molecular ratios, with the shellac content ranging from 10% to 30% in the final reaction mixture (as shown in Figure 3b,c). Additionally, different doses of gamma radiation, ranging from 10 to 50 kGy, were applied during the synthesis process [54,55,64].

Bio-based hydrogels, denoted as PC-PAAc/GA, were synthesized using gamma irradiation to remove lead cations from simulated solutions. These hydrogels consisted of pectin (PC) and polyacrylic acid (PAAc), reinforced with different ratios of gallic acid (GA) (as shown in Figure 3). The irradiation dose applied was 20 kGy. The experimental data revealed that swelling increased with the pH of the medium, reaching equilibrium after 350 min. Interestingly, the maximum swelling was achieved at a pH level of 10 for both PC-PAAc and PC-PAA/GA1.5 formulations (as shown in Figure 3d) [65].

A parallel investigation into the creation of potato starch/acrylic-acid hydrogels via gamma radiation underlines an innovative pathway to produce materials with superior absorption qualities. Such hydrogels exhibit outstanding performance in dye adsorption, positioning them as viable candidates for eco-friendly water purification methods. This methodology capitalizes on gamma radiation’s crosslinking strengths alongside the inherent polysaccharide framework of potato starch, further augmented by acrylic acid, to effectively purify water by eliminating pollutants [66,67,68].

**Figure 4:**
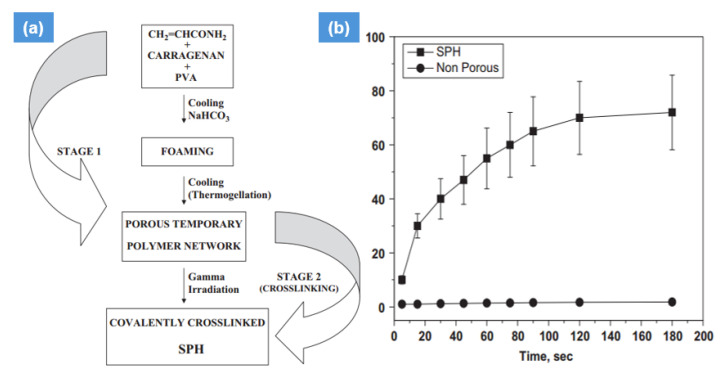
(**a**) Steps in creating polyacrylamide SPH and (**b**) swelling kinetics of SPH and conventional non-porous polyacrylamide hydrogels, reproduced with permission from ref. [70]. Copyright, Elsevier.

A scientific investigation explored the creation of various hydrogel structures using gamma radiation, combining chitosan and N,N-dimethylacrylamide. The research delved into modifying chitosan with DMAAm in three distinct architectures: comb-type grafting hydrogels (net-CS)-g-DMAAm, interpenetrating networks of CS and DMAAm (net-CS)-inter-(net-DMAAm), and semi-interpenetrating networks (net-DMAAm)-inter-CS. These different polymer configurations were produced via gamma irradiation from a 60Co source. The degree of crosslinking notably increased with both DMAAm concentration and radiation dosage, achieving 80% crosslinking at a 10% *v*/*v* concentration and nearly full crosslinking at higher concentrations (as shown in Figure 4). It was observed that a minimum dose of 3 kGy was adequate for the complete crosslinking of the DMAAm, beyond which an additional dosage only amplified the crosslinking density. Various methods have been employed to create these structures [69,70].

**Figure 5:**
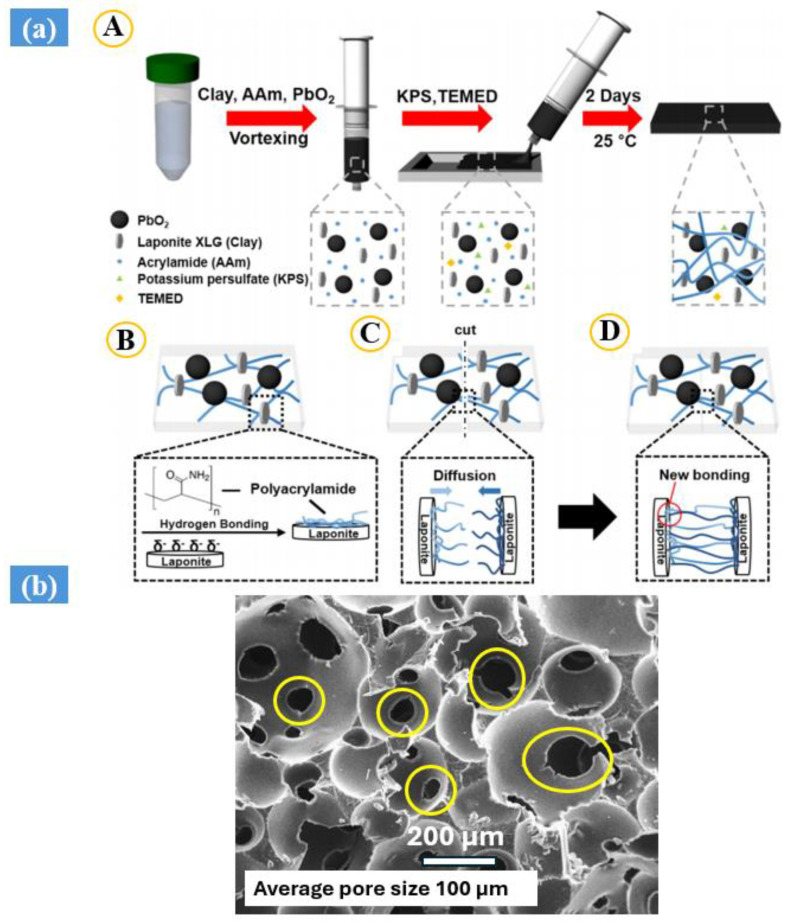
(**a**) (A) Fabrication of a stretchable, self-healing hydrogel–metal oxide composite for γ-ray radiation. (B) Diagram showing polyacrylamide chains hydrogen-bonded to Laponite within hydrogel composites. (C,D) Formation of new hydrogen bonds between cut polyacrylamide chains and Laponite via thermal diffusion [38]. (**b**) SEM of polyacrylamide SPH, reproduced with permission from ref. [70]. Copyright, Elsevier.

The method for creating super porous hydrogels (SPHs) using gamma radiation has been outlined in 2009. Unlike traditional SPH synthesis, which has combined foaming and crosslinking simultaneously, radiation synthesis has faced challenges in coordinating these processes. To address this, the foaming and radiation crosslinking stages have been split into two steps. This method has yielded a polyacrylamide SPH with rapid swelling kinetics, showing a significant improvement over non-porous polyacrylamide hydrogels. The SPH has achieved an equilibrium swelling of around 70 g/g in just 2 min, in contrast to the conventional hydrogel, which has reached only 1.7 g/g in the same time frame. The porous structure, with an average pore size of 100 μm, has been examined using scanning electron microscopy (SEM) after dehydration and air drying (as shown in Figure 5). This separation of processes has allowed for the successful creation of super porous hydrogels with enhanced swelling properties, presenting a promising advancement in hydrogel synthesis (as shown in Figure 4a,b) [70,71]. Other research innovations include the development of a self-healable soft shield designed for protection against gamma ray radiation. This shield was based on polyacrylamide hydrogel composites, demonstrating an advanced approach to shielding technology. By exploring the unique properties of polyacrylamide hydrogels and their self-healing capabilities, the research aimed to provide a versatile and efficient solution for shielding against gamma ray radiation exposure [38].

It has been studied that gamma irradiation was employed to fabricate hydrogels that combine α-cellulose with cellulose nanocrystals (CNCs) within gelatin in the absence of crosslinking agents. During the production process, alkali and bleaching treatments were conducted on rice husks to extract cellulose from them, followed by acid hydrolysis so that CNCs could be produced [72,73,74]. The compatibility between gelatin and the cellulosic materials was then exploited to form a semi-interpenetrating network of hydrogels. The stiffness and swelling properties of hydrogels created by dispersing CNCs were significantly better than those created by dispersing α-cellulose. It was concluded that the uniform distribution of CNCs throughout the gel matrix and their increased crystallinity played a significant role in this improvement.

### 2.3. Viscoelastic Properties of Radiation-Induced Hydrogels

Recent developments in radiation-induced hydrogels have notably emphasized the role of viscoelastic properties and their crucial impact on the performance and suitability of these materials for a range of biomedical applications. Viscoelasticity, which integrates both viscous and elastic characteristics, is primarily determined by the degree and nature of crosslinking achieved through ionizing radiation, such as gamma or electron beams. As the dose of radiation increases, there is an enhancement in the crosslink density within the hydrogel network. This increment generally improves the material’s elastic modulus, leading to firmer hydrogels that offer superior structural support, a property highly valued in applications such as cartilage regeneration and various forms of tissue engineering scaffolds [75]. The influence of the radiation dose on the viscoelastic properties, however, extends beyond just increasing firmness. Higher-density crosslinking enhances the viscous properties, enabling these hydrogels to better absorb and dissipate mechanical energy, which is vital for applications involving dynamic mechanical stress such as joint movement or during the pulsatile flow of blood. This capability is essential for maintaining the integrity and functionality of the hydrogel under physiological conditions [76]. Furthermore, the specific chemical composition of the polymers employed plays a significant role in defining the viscoelastic properties. The mechanical strength and biocompatibility of hydrogels are affected by the interactions between synthetic polymers like polyethylene glycol, as well as natural polymers like alginate and chitosan when exposed to radiation. For instance, alginate-based hydrogels have shown considerable promise due to their inherent biocompatibility and the ability to fine-tune their mechanical properties through controlled irradiation [77]. Recent innovations also include the integration of nano-additives into these hydrogels, a move that has significantly bolstered both mechanical properties and functional capabilities. Metallic nanoparticles, for example, not only enhance mechanical robustness but also introduce additional functionalities like electrical conductivity and improved thermal stability. These properties are invaluable in more complex applications such as biosensors and actuators [78]. The nano-additives also help tailor the hydrogels’ response to environmental factors such as pH, temperature, and ionic strength, which is key for developing targeted drug delivery systems that are responsive to specific physiological triggers. The ability to manipulate the viscoelastic properties through an innovative combination of polymer chemistry, radiation crosslinking techniques, and nano-additives opens up new opportunities in the design and application of radiation-induced hydrogels. The deep understanding and further exploration of these properties are critical, as they influence not only the theoretical design but also the practical implementation of these hydrogels in medical applications, where their demand is steadily increasing. Research in this field will continue to push the limits of these versatile materials, utilizing their unique properties to tackle complex challenges in healthcare and beyond [79].

## 3. Electron Beam Irradiation Synthesis

Electron beam radiation plays a vital role in hydrogel synthesis as it effectively starts polymerization events and facilitates the crosslinking of polymer chains. This process results in the creation of a strong and durable three-dimensional network structure, which is crucial for the hydrogel’s mechanical strength and durability [35]. This process allows for precise control over the physical, chemical, and biological properties of the hydrogel by adjusting parameters such as dose, beam energy, and irradiation time, enabling tailored modifications to meet specific application requirements in areas like tissue engineering, drug delivery, and wound healing [36]. Electron beam radiation represents a powerful tool for the precise modification and enhancement of materials, offering tailored properties that find application across various sectors. In biomedical research, the electron beam crosslinking of hydrogels, such as polyethylene glycol diacrylate, enables the creation of scaffolds with tunable mechanical properties and biocompatibility, crucial for tissue engineering applications that require adequate support for cell growth and tissue regeneration [80]. Furthermore, in the realm of food packaging, the electron beam irradiation of polymers like polyethylene terephthalate (PET) enhances barrier properties against gases and moisture, extending the shelf life of packaged products and ensuring food safety by preventing contamination [81]. In environmental engineering, the electron beam treatment of hydrogels functionalized with specific groups, such as acrylic acid, allows for the efficient removal of organic pollutants from contaminated water systems through enhanced adsorption capacities and tailored chemical interactions [82]. These specific examples underscore the versatility and efficacy of electron beam radiation in tailoring material properties to meet the demands of targeted applications, showcasing its pivotal role in advancing diverse industries.

The versatility of electron beam radiation as a tool for customizing hydrogel properties highlights its significance in advancing materials for biomedical and industrial applications, offering researchers a sophisticated means to manipulate and optimize hydrogel characteristics [83]. It has several advantages:

1. Electron beam (EB) radiation is an environmentally friendly process that does not require chemicals, ensuring a clean and sustainable treatment method.
2. EB radiation can uniformly penetrate materials deeply, enabling the precise sterilization and modification of substances.
3. This technology is rapid, cost-effective, and easily scalable for industrial production, providing efficiency in various applications.
4. EB radiation leaves no harmful residues or by-products, ensuring the safety and purity of treated materials.
5. The controlled processing parameters of EB radiation allow for customizable outcomes in fields such as healthcare, food preservation, and materials science.

**Figure 6:**
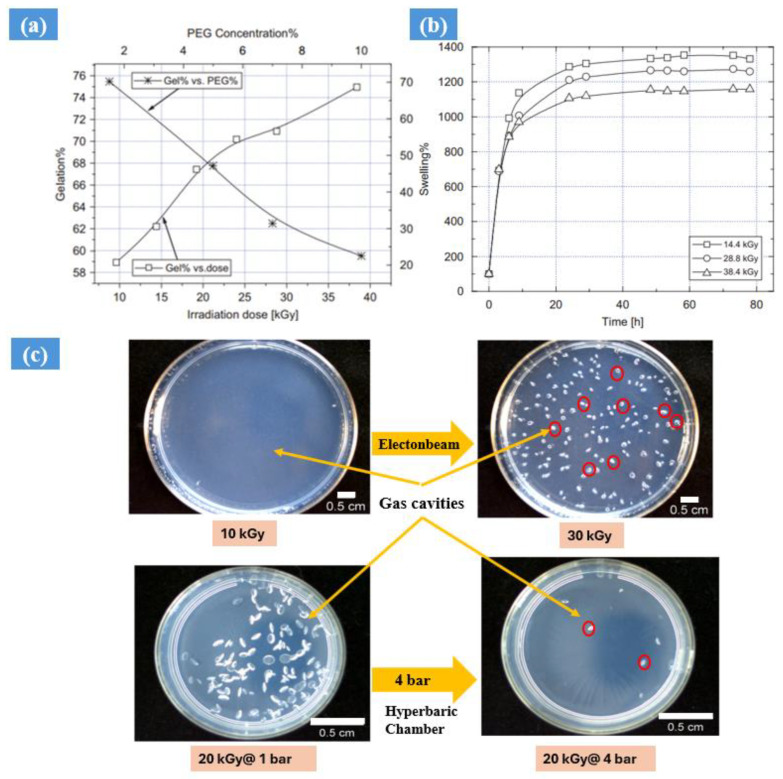
(**a**) Relation between gel fraction% in dressings and irradiation dose (PVP = 8%) and PEG concentration (25 kGy). (**b**) Time-dependent swelling kinetics of hydrogel dressings at various doses, reproduced with permission from ref. [84] copyright, Elsevier. (**c**) Images of 1 wt.% agarose hydrogels after irradiation at 10 kGy, 30 kGy, and 20 kGy under 1 bar, and 4 bar pressure, reproduced with permission from ref. [86] copyright, Elsevier.

A hydrogel wound dressing that contains PVP, PEG, and agar was produced using electron beam technology with the aid of an electron accelerator. The research involved an investigation into various process parameters to tailor the properties of the hydrogels. The study revealed that the gel fraction percentage rose with an increase in irradiation dose but decreased with a higher PEG content (as shown in Figure 6a). Conversely, the maximum swelling percentage decreased with a higher irradiation dose while increasing alongside higher PEG concentrations (as shown in Figure 6b). Notably, PEG played a significant role in altering both the gel fraction percentage and maximum swelling percentage in response to the irradiation dose. Additionally, the developed dressings were shown to serve as effective barriers against microbes, further highlighting their potential utility in wound care applications [56,57,84,85]. Attempts were made to explore the effects of high-energy electron irradiation on agarose hydrogels, looking at how it affects their properties in terms of their physical, structural, and chemical characteristics. Approximately 30 kGy of sterilization doses were used in this study.

Following irradiation, gas cavities formed within the hydrogels, increasing in both quantity and size with higher doses. Images taken after irradiating at 10 kGy and 30 kGy, revealing prominent gas cavities. The researchers observed that crosslinking in an autoclave at a pressure of 5 bar prevented gas cavity formation by enhancing the CO_2_ solubility in water. Similarly, during electron beam treatment under hyperbaric conditions at a pressure of 4 bar, a decrease in the formation of gas cavities was observed (as shown in Figure 6c) [86,87].

In a study, polybutylene terephthalate (PBT) was irradiated with electron beams and treated with halogen-free flame retardants to determine how they affected the properties of the material. The irradiation was performed using a Rhodotron TT200 10 MeV electron beam in air, with samples receiving total absorbed doses of 200 to 400 kGy at room temperature. The results showed that electron beam exposure improved the polymer’s strength and hardness, while the incorporation of flame retardants led to a decrease in mechanical properties. Additionally, irradiating the polymer led to a reduction of the dielectric loss coefficient, as well as the formation of char and the increase in residual char content after the irradiation process [88,89].

**Figure 7:**
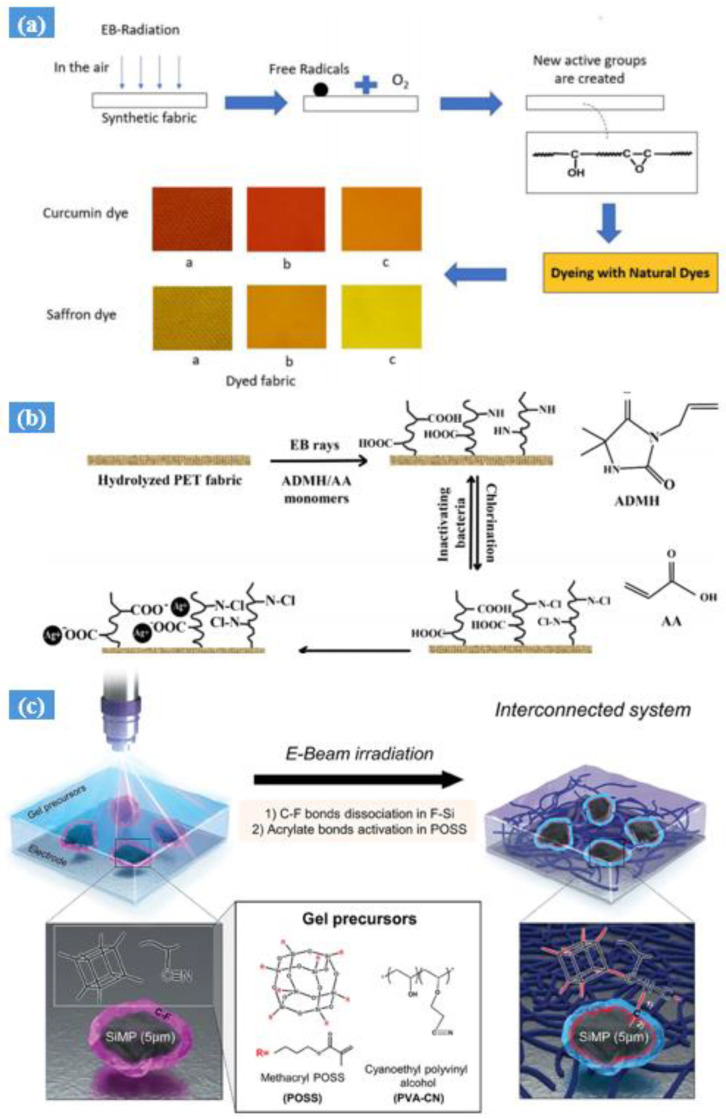
(**a**) EBI dyeing mechanism for treated synthetic fabrics (a: PP, b: Nylon 6. c: PET) and (**b**) an overview of the process for preparing hydrophilic and antibacterial PET fabrics [90]. (**c**) A schematic illustration showing the in situ formation of covalent links between silicon microparticle anodes and multifunctional gel polymer electrolytes using electron beams [100].

A review article has been published on the surface modification of textiles by electron beam irradiation recently by Abou Elmaaty et al. [90]. In their study, natural dyes such as curcumin and saffron were pretreated with electron beam irradiation (EBI) prior to dyeing polypropylene (PP), nylon 6, and polyethylene terephthalate (PET). These synthetic fabrics were examined to determine whether they would be affected by exposure doses ranging from 0 to 300 kGy and the duration of the oxidation process in the air at these exposures. The study highlighted that the optimal conditions were attained at 300 kGy with a one-hour oxidation time grafting with the N-halamine precursor monomer acrylic acid (AA) and 3-allyl-5,5-dimethyl hydantoin (ADMH), followed by irradiation with electron beam radiation (EBI), has been identified as an effective and environmentally friendly approach to modify PET fabric for improved antibacterial and wettability properties [91]. In this method, a highly hydrophilic surface was achieved by pre-treating PET fabric with alkali and grafting on a hydrophilic monomer (as shown in Figure 7a,b). The combination of these techniques presents a promising avenue for enhancing the functionality and performance of PET textiles in antibacterial applications and moisture management [92,93,94].

An alternative study revealed that hydrogels produced via electron beam polymerization showcased superior mechanical attributes and optical clarity when contrasted with traditional UV-cured hydrogels. Noteworthy enhancements included heightened elasticity, increased crosslinking density, and enhanced transparency spanning a broader range of wavelengths. The investigation meticulously scrutinized the interplay between mechanical and optical properties concerning differing single differential and overall irradiation doses. These hydrogels were purposefully engineered for potential deployment in drug delivery applications, with methylene blue serving as the prototype drug model [83,95,96,97,98,99]. These hydrogels were purposefully engineered for potential deployment in drug delivery applications with a prototype drug model; methylene blue was used.

**Figure 8:**
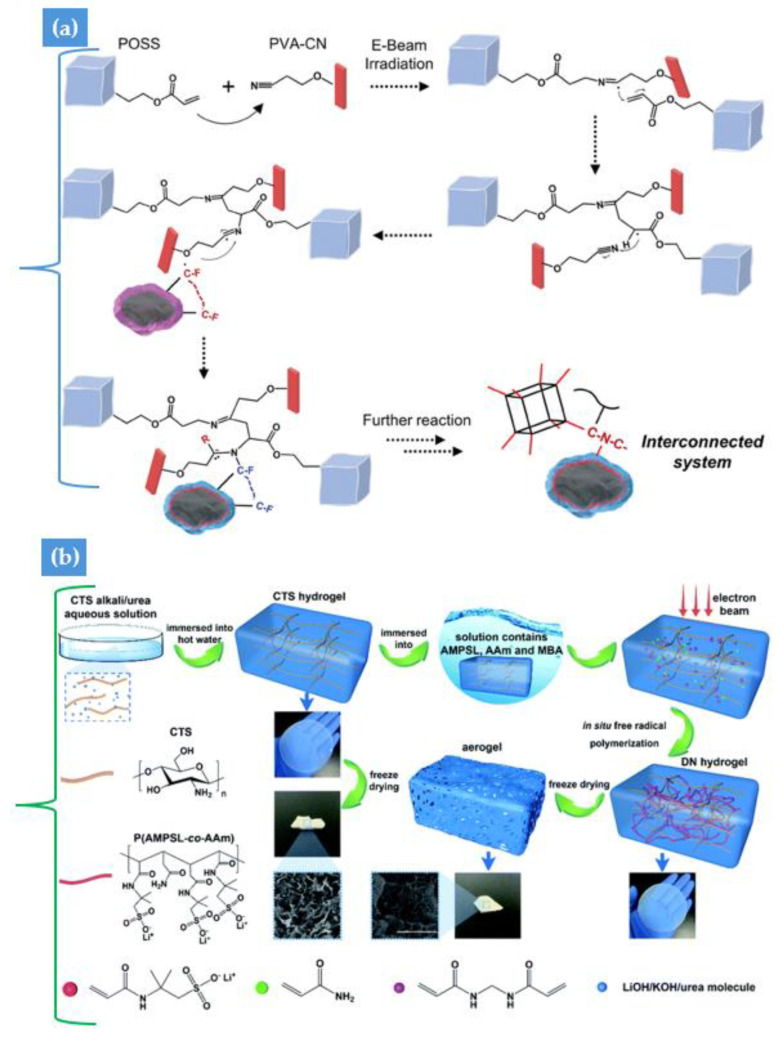
(**a**) Proposed mechanism for the crosslinking reaction via electron beam irradiation [100]. (**b**) Schematic of synthesis for chitosan/P(AMPSL-co-AAm) hydrogel and aerogel initiated using electron beam radiation [101].

A novel system was developed by integrating large silicon microparticles (SiMPs) with a multifunctional gel polymer electrolyte (GPE) using electron beam exposure. This process created an intertwined gel system with excellent properties, showcasing effective stress dissipation and high ionic conductivity. The system’s design offers potential advancements in energy storage technologies for next-generation batteries (as shown in Figure 7c and Figure 8a) [100].

## 4. UV Radiation Hydrogel Synthesis

It was found that ultraviolet (254 nm) radiation can create a thermally stable substrate to produce cell scaffolds using a gelatin–glucose matrix. Using differential scanning calorimetry, the research found that UV irradiation substantially boosted the thermal stability of the gelatin–glucose hydrogels, preventing any melting at temperatures up to 90 °C. The glucose addition not only increased the crosslinking yield but also played a crucial role in the crosslink formation, as further evidenced using scanning electron microscopy, which exposed a distinct density variation in the irradiated samples, highlighting the structural changes due to UV exposure [58,102]. The use of UV radiation in hydrogel synthesis presents a groundbreaking methodology that allows for precise control over the structural properties and functionalities of the resulting polymeric networks. Exposing monomers and crosslinkers to UV light in the presence of photo-initiators initiates polymerization reactions, resulting in hydrogels with tailored mechanical strength, biocompatibility, and responsiveness. UV-induced hydrogels have become important materials in biomedical engineering for advanced wound healing applications. These hydrogels are very good at dressing wounds because they create the right conditions for tissue regeneration and controlled drug release, which speeds up the healing process and lowers the risk of complications [59,103]. Furthermore, recent studies have demonstrated the efficacy of UV-induced hydrogels in agricultural applications [7], where they contribute to improved soil hydration, enhanced seed germination rates, and increased crop yields, thus addressing key challenges in sustainable agriculture. Additionally, in environmental management, UV-induced hydrogels have shown remarkable potential for water purification, highlighting their capacity to effectively remove pollutants from water sources. Notably, UV-induced hydrogels are excellent at absorbing oil spills and removing them from the environment, making them a long-term solution for environmental cleaning. The synthesis of hydrogels through UV radiation has significantly impacted the field of agriculture by offering novel solutions to address key challenges in crop cultivation and soil management. By leveraging the unique properties of UV-induced hydrogels, researchers and agricultural practitioners have unlocked innovative approaches to enhance agricultural productivity and sustainability. Researchers have shown that encapsulating agricultural inputs like fertilizers and nutrients within UV-induced hydrogels enhances nutrient availability for plants, reduces nutrient leaching, ensures efficient nutrient uptake, and minimizes environmental nutrient losses. Furthermore, the application of UV-induced hydrogels in seed coating technologies has revolutionized seed germination and early-stage plant growth. The utilization of UV radiation-induced hydrogels in food safety practices represents a critical advancement in ensuring the quality and integrity of food products throughout the production and distribution processes. UV-induced hydrogels offer unique benefits that can enhance food safety measures in several key areas. For instance, the integration of these hydrogels into food packaging materials can enhance the shelf life of perishable items by incorporating antimicrobial agents or oxygen scavengers, which inhibit bacterial growth and reduce oxidation. In addition, UV-induced hydrogels can be tailored for the selective capture and detection of food contaminants, providing a sensitive detection mechanism for pathogens, toxins, and chemical residues in food samples [104]. UV-induced hydrogels facilitate water purification in food processing facilities, effectively removing pollutants and contaminants from water sources to ensure the safety and quality of water used in food production processes. By controlling the release of food additives, preserving foods with edible coatings, and improving food packaging, UV radiation-induced hydrogels offer a comprehensive approach to enhancing food safety across the food industry. Hydrogels synthesized using light-sensitive functional groups offer significant benefits, such as simplicity, rapid preparation, and lower production costs compared to chemical crosslinking.

## 5. Brief Application of Hydrogels

### 5.1. Pharmaceuticals Application

Radiation-induced hydrogels are specialized materials formed by the crosslinking of polymers using radiation, such as gamma rays or electron beams. These hydrogels exhibit unique properties, including high water content, biocompatibility, and controlled mechanical strength, which make them particularly valuable in pharmaceutical applications. A key application is in the development of advanced drug delivery systems. These hydrogels can be engineered to encapsulate a drug and release it in a controlled manner, enhancing therapeutic efficacy while minimizing side effects. For instance, polyvinylpyrrolidone (PVP)-based hydrogels, crosslinked with gamma radiation, have been shown to manage insulin release effectively, providing prolonged and stable glucose regulation for diabetic patients [105]. Additionally, radiation-induced hydrogels are making strides in gene therapy. Polyethylene glycol (PEG)-based hydrogels have been utilized as carriers for DNA and RNA, enabling the controlled release of genetic material into specific cells. This method shows promise for treating genetic disorders and for cancer immunotherapy by modifying the genetic expression within targeted cells [106]. Another unique application is in the development of hydrogels as biosensors for the real-time monitoring of physiological conditions. For instance, hydrogels that incorporate fluorescent or electrochemical sensors can be used to detect changes in glucose or pH levels, providing continuous and non-invasive monitoring for diabetic patients [107]. The porous structure of hydrogels serves as an effective matrix for drug loading, providing protection from adverse environmental conditions while facilitating drug delivery. The porosity of the gel matrix can be tailored by manipulating the crosslinking density. Moreover, the pace at which pharmaceuticals are released, which is a crucial aspect for drug delivery systems, is mainly determined by the diffusion coefficient of the molecule across the gel network and can therefore be customized to fulfill precise requirements. Hydrogels can be optimized to enhance their suitability for drug delivery applications by achieving biocompatibility and biodegradability through the design of specific physical and chemical structures. These characteristics underscore the significant potential of hydrogels as versatile drug delivery systems [108,109].

This study explores the preparation of ferrogels through the incorporation of iron oxide nanoparticles into porcine gelatin using electron beam assistance. The resulting bio ferrogels demonstrate potential for diverse applications, including tissue engineering, soft actuation, and controlled drug release. By combining biocompatible components with magnetic responsiveness, these materials offer a promising avenue for developing mechanical transducers that are contactless and can be used in vivo [110,111]. A recent study introduced a chitosan/lithium sulfonate double network hydrogel/aerogel designed for efficient CO_2_ capture, aiming to address carbon emissions. Utilizing electron beam radiation, the hydrogel’s uniform and rapid polymerization sets it apart from traditional methods. The resulting aerogels display excellent physical and chemical stability with a porous structure ideal for CO_2_ capture. This cost-effective approach demonstrates promising applications in the development of CO_2_ solid adsorbents (as shown in Figure 8b) [101].

In 2017, an investigation assessed the synthesis of hydrogels through electron beam irradiation for the purpose of heavy metal adsorption. Polyacrylamide co-acrylic acid hydrogels were produced using the free-radical copolymerization of acrylamide and acrylic acid in aqueous solutions. The irradiation process was conducted at room temperature in atmospheric conditions, with doses ranging from 2.5 kGy to 6 kGy. Researchers explored how varying the absorbed dose, as well as the amounts of crosslinker (trimethylolpropane trimethacrylate) and initiator (potassium persulfate), impacted the swelling properties, diffusion coefficient, and network parameters of the hydrogels [112,113].

### 5.2. Biomedical Engineering

#### 5.2.1. Skin Care

**Figure 9:**
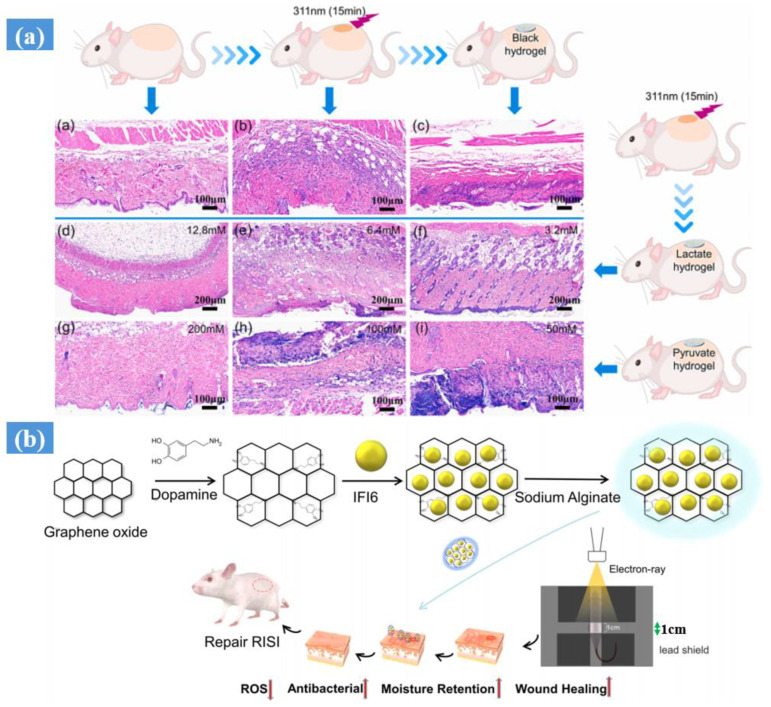
(**a**) H and E staining images of a UV-irradiated BALB/c mouse skin model treated with composite hydrogel films: (a) normal skin, (b) 311 nm UV-irradiated skin, (c) hydrogels without Pyruvate and lactic acid, (d–f) hydrogels with different lactate concentrations (12.8, 6.4, and 3.2 mM), and (g–i) hydrogels with varied pyruvate levels (200, 100, and 50 mM); reproduced with permission from ref. [114]. Copyright, Elsevier. (**b**) An illustration of a schematic diagram showing how the IFI6-PDA@GO/SA is fabricated and how it is used to heal RISIs (radiation-induced skin injuries) [115].

In 2023, M. Liu et al. studied the efficacy of hydrogel films based on pyruvate and lactate in mitigating UV radiation-induced skin inflammation and oxidative stress. The study focused on integrating lactic and pyruvic acids into a hydrogel to serve as a topical treatment for solar dermatitis, catering to both free-radical scavenging and inflammation modulation. The research provided detailed schematic illustrations of the gel components and the application process for treating UV-damaged skin. This approach was thoroughly validated to confirm the effectiveness of combining lactate and pyruvate in treating UV-induced skin photodamage, indicating a significant potential for clinical application. The results from the experiments, including images stained with H and E staining taken from a UV-irradiated BALB/c mouse skin injury model treated with the composite hydrogel film, showed that inflammation was reduced in the presence of this composite hydrogel film. The hydrogels, containing varying concentrations of lactate (12.8, 6.4, and 3.2 mM) and pyruvate (200, 100, and 50 mM), showed progressively fewer inflammatory cells, highlighting their potential therapeutic benefits through their self-tissue-repairing mechanism (as shown in Figure 9a) [114]. Another promising application of IFI6 involves promoting the healing of radiation-induced skin injuries (RISI) by modulating HSF1 activity. A sprayable composite hydrogel containing IFI6-PDA@GO/SA was developed for use with HaCaT skin cells, which was shown to enhance proliferation and migration, which provided synergistic radio resistance both in vitro and in vivo. Additionally, the study evaluated the biological activity of IFI6 in wound healing using these hydrogels for skin regeneration, assessing cell proliferation, migration, and angiogenesis. This research highlights the significant potential of IFI6-based treatments in managing and healing RISI, advocating for further investigation into its broader therapeutic applications (as shown in Figure 9b) [115,116,117].

**Figure 10:**
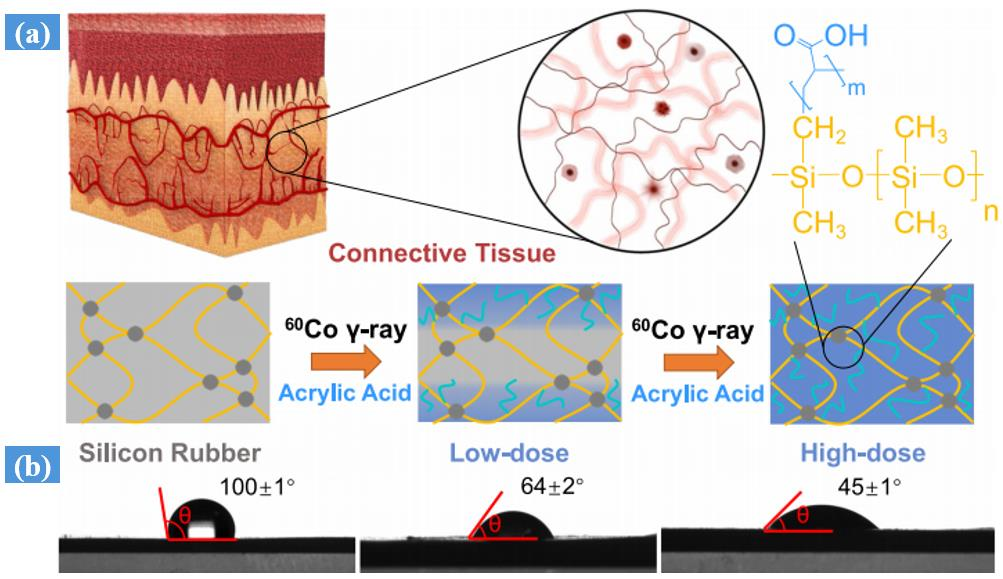
Concept design and structural analysis of CEBH: (**a**) schematic illustrating silicone rubber modification into CEBH using acrylic acid. (**b**) Experimental optical diagram showing contact angle variations of modified silicone rubber with different AC [118].

Yunlong Wang’s research team has developed an innovative technique for creating artificial skin using an elastomer-based hydrogel, inspired by the properties of connective tissue. Utilizing a one-step radiation-induced penetrating polymerization process, the group successfully transformed commercial silicone rubber into connective-tissue-inspired elastomer-based hybrids (CEBHs). This approach is pivotal for their potential use in biomedical applications, particularly as artificial skins. The resulting CEBH demonstrates outstanding mechanical strength, ion sensitivity, and adhesion properties comparable to human skin, making it a promising material for various medical applications (as shown in Figure 10) [118].

#### 5.2.2. Cancer Therapy

Gamma ray-synthesized hydrogels have emerged as a promising tool in cancer therapy, offering notable benefits in drug delivery and radiation therapy applications [119]. These hydrogels are fabricated through gamma irradiation-induced polymerization, allowing for the precise delivery of anti-cancer agents directly to the tumor site. By encapsulating chemotherapy drugs or radioisotopes, gamma ray-synthesized hydrogels enable the targeted and controlled release of therapeutic payloads, enhancing treatment efficacy while minimizing systemic toxicity and adverse effects on healthy tissues. The tunable properties of these hydrogels permit the customization of drug release kinetics, providing flexibility in tailoring treatment regimens to individual patient needs. Moreover, their capability to encapsulate and deliver radioisotopes for radiation therapy offers a synergistic approach that can improve treatment outcomes by increasing the effective radiation dose delivered to the tumor while sparing surrounding healthy tissues [120]. Minhas et al. developed a degradable hydrogel-based device targeting the colon for the oral delivery of 5-FU [121]. They synthesized ethylene glycol dimethacrylate (EGDMA)-crosslinked hydrogels and modified them with methacrylic acid (MAA) to ensure pH responsiveness and benzoyl peroxide (BPO) for crosslinking polymerization. The results showed the high potential of the pectin-co-poly(MAA) hydrogels for the targeted delivery of 5-FU with negligible exposure of the upper gastrointestinal tract. Sung In Jeong et al. studied the one-step synthesis of a gene carrier via gamma irradiation and its application in tumor gene therapy [122]. They revealed the selective grafting of AEMA onto C6-OH groups of WSC. AEMA-g-WSC effectively condensed plasmid DNA to form polyplexes in the size range of 170 to 282 nm. AEMA-g-WSC polyplexes, in combination with psi-hBCL2 (a vector expressing short hairpin RNA against BCL2 mRNA), inhibited tumor cell proliferation and tumor growth in vitro and in vivo, respectively, by inducing apoptosis.

Hydrogels have emerged as exceptional materials in medical science, noted for their biodegradability, biocompatibility, and capability to manage drug release effectively. These properties make them highly valuable in various cancer treatment modalities, such as chemotherapy, radiotherapy, immunotherapy, and more innovative approaches like photodynamic and photothermal therapies. As an adjunct or a primary treatment in chemotherapy, hydrogels address common challenges such as non-specific targeting, severe side effects, and poor drug tolerance that are typical of traditional chemotherapy drugs [123]. By forming through crosslinking polymerization in aqueous solutions, hydrogels prevent drug denaturation and aggregation, enhancing the drugs’ efficacy while reducing adverse reactions and improving systemic tolerance.

Innovatively, J.H. Lee et al. [124] developed a hydrogel responsive to temperature and pH variations, which precisely modulates anti-cancer drug release, achieving significant cell mortality while potentially reducing side effects. Similarly, hydrogels are making strides in radiation therapy, typically used to disrupt cancer DNA and shrink tumors by creating a more distributive platform for radionuclides. This function, combined with the ability of hydrogels to contain radiosensitizers, directly addresses the inherent challenges of radiation resistance and incomplete DNA damage repair encountered in solid tumors. N. Wang et al. [125] demonstrated the application of a hydrogel formulated from endostatin and hyaluronic acid–tyramine, which effectively modulated the tumor environment to enhance radiation sensitivity. Further contributing to this area, J. Zhang et al. [126] crafted a unique multifunctional hydrogel that integrates radiosensitizers like gold nanoparticle aggregates, along with drugs such as doxorubicin and radiolabeled iodine-131. This innovative hydrogel design not only optimizes radiation therapy but also creates a synergistic treatment platform that could significantly expand the efficacy of cancer treatments. These advancements illustrate the pivotal role of hydrogels in transforming cancer therapies, offering more precision, reduced side effects, and enhanced efficacy in combating various cancer stages [120,127]. Hydrogels continue to stand out as a cornerstone in the development of next-generation therapeutic solutions in oncology, highlighting their indispensable role in evolving chemotherapy treatment paradigms.

#### 5.2.3. Drug Delivery System

The utilization of radiation-induced hydrogels for drug delivery systems is considered an innovative and promising approach in the biomedical field. These hydrogels, synthesized using radiation methods like gamma irradiation, possess tailored properties that make them well-suited for controlled drug release [128]. In a study conducted by Baljit Singh et al., hydrogels developed through radiation-induced polymerization were employed for enhancing the drug release of indinavir sulfate, a potent HIV protease inhibitor. These hydrogels, prepared using dietary fiber psyllium and a mixture of acrylamide (AAm) and 2-acrylamido-2-methylpropanesulfonic acid (AMPSA), showcased potential as controlled drug delivery systems [129]. Similarly, M. Carenza et al. explored the use of hydrogels obtained through radiation-induced polymerization as delivery systems for peptides and protein drugs. They observed that the controlled release of peptides and proteins from these hydrogels, produced via the radiation-induced polymerization of 2-hydroxyethyl methacrylate, varied based on factors such as protein molecular weight and the presence of polyethylene glycol (PEG) during polymerization. The study highlighted the influence of polymer matrices’ swellability and porosity on the release kinetics of peptides and proteins [130].

Global efforts have ramped up towards refining drug delivery systems that provide controlled dosages over extended durations within the targeted areas [131]. The key structural requirements of an efficient drug delivery system include a drug storage region, controlled release capability, and a release mechanism [132]. Notably, hydrogels offer all three functions and possess the ability to mask the unpleasant taste and odor often associated with pharmaceuticals. Due to these versatile properties, hydrogels find wide-ranging application in oral, nasal, buccal, rectal, vaginal, ocular, injectable, and various other administration routes [47]. Once hydrogels are introduced into the body, they serve to ensure the controlled release of embedded drugs into bodily fluids. In addressing challenges associated with lipophilic drugs such as poor solubility, dispersion inconsistencies, and limited bioavailability, the integration of these drugs into hydrogel systems offers a solution, enhancing drug solubility, stability, and bioactivity while enabling sustained or controlled drug release. Conversely, while highly soluble small molecule drugs offer benefits like improved absorption and bioavailability, they are less suited for sustained delivery effects. To capitalize on the advantageous properties of both types of drugs, a novel interpenetrating polymer network was formulated by modifying silicone elastomers with a poly(2-hydroxyethyl methacrylate) (PHEMA)-based hydrogel acting as a hydrophilic carrier within the silicone network structure, effectively embedding the antibiotic ciprofloxacin. Consequently, these systems demonstrate potential for use in the development of drug-releasing medical devices [133].

C. Liu and colleagues worked on enhancing drug carrier capacity and achieving the sustained release of the anticancer drug methotrexate (MTX) by developing gelatin-based hydrogels using β-cyclodextrin (β-CD) as a crosslinking agent. The hydrogel β-CD-Gel-3, containing 15.2% by weight of β-CD, demonstrated the highest MTX loading capacity at 16.4 mg per gram of the hydrogel. Comparatively, hydrogels with 11.1% or 13.5% β-CD content could retain 12.2 mg and 14.9 mg of MTX per gram of hydrogel, respectively. This study also involved a dextran-crosslinked gelatin-based hydrogel for comparison [134].

### 5.3. Application of Hydrogel: In Agriculture

Radiation-induced hydrogels, created through gamma irradiation, represent an innovative approach in agriculture by providing a controlled release mechanism for essential agricultural inputs like fertilizers and pesticides [135]. These hydrogels effectively optimize nutrient delivery to plants, improve soil moisture retention, and contribute to sustainable farming practices. By facilitating a gradual and targeted release of nutrients, radiation-induced hydrogels offer a practical solution for enhancing crop productivity while reducing environmental impact through minimized leaching and improved soil health management [136]. This technology holds significant promise for revolutionizing agricultural practices towards a more efficient and eco-friendly farming system.

A study conducted by A. I. Raafat et al. [137] investigated the radiation synthesis of superabsorbent hydrogels using carboxymethylcellulose (CMC) and polyvinylpyrrolidone (PVP) crosslinked with gamma irradiation for agriculture applications. The research showed that the composition and irradiation dose significantly affected the swelling degree of the CMC/PVP hydrogels, which were designed to gradually release nutrients, with urea used as an agrochemical model to provide nitrogen nutrients. Another study by Ahmed M. Elbarbary et al. [138] explored the radiation-induced crosslinking of polyacrylamide (PAAm) incorporated with low-molecular-weight natural polymers, such as Na-alginate (Alg) or chitosan (CS), for potential agricultural applications. These superabsorbent hydrogels, synthesized using γ-rays, demonstrated positive effects on the growth and yield of maize plants when used in agricultural fields. Notably, maize plants treated with PAAm/Alg hydrogels showed a 50% increase in grain yield, indicating the potential of these hydrogels as soil conditioners and water reservoirs in plant–soil systems.

**Scheme 1:**
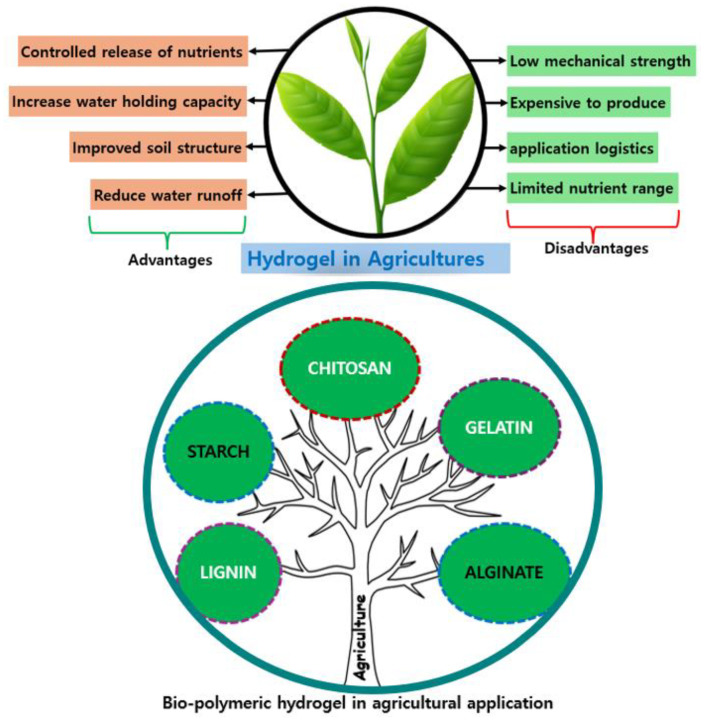
Biodegradable hydrogels in agriculture: exploring the benefits and drawbacks of bio-polymeric solutions.

Hydrogels play a vital role in modern agriculture by addressing various challenges related to water management and nutrient delivery. One of the key functions of hydrogels in agriculture is their exceptional water retention capacity. These polymers have the ability to absorb and retain significant amounts of water, forming a gel-like structure that can slowly release moisture to plant roots over time. This property is particularly advantageous in regions prone to drought or water scarcity, as hydrogels can help maintain soil moisture levels and reduce the frequency of irrigation, thereby conserving water resources [7]. Moreover, hydrogels serve as effective carriers for nutrients and fertilizers in agriculture. By encapsulating nutrients within their structure, hydrogels can facilitate controlled release mechanisms, ensuring that plants receive a steady and consistent supply of essential nutrients. This controlled delivery system not only enhances nutrient uptake by plants but also minimizes nutrient loss through leaching or runoff, promoting more efficient nutrient utilization and reducing environmental impact [139]. In addition to their water retention and nutrient delivery functions, hydrogels can also improve soil structure and aeration. By adding hydrogels to soil, farmers can enhance soil aggregation, prevent soil compaction, and promote root development. This, in turn, leads to improved soil fertility, better plant growth, and increased crop yields [140]. The common bio-polymeric hydrogels and their advantages and disadvantages are shown in Scheme 1.

Overall, the use of hydrogels in agriculture contributes to sustainable farming practices, water conservation, efficient nutrient management, and enhanced crop productivity. Their versatile applications make them valuable tools for modern agricultural systems seeking to address the challenges of changing climates and growing food demand.

In the field of urban agriculture, advancements involving hydrogels have garnered attention over recent decades as urban farming systems have evolved to manipulate light, nutrient solutions, and the plant growth medium. Water sourcing, a critical aspect essential for plant growth, remains a major focus of study. One notable innovation in urban agriculture is the integration of hydrogels as a crucial component in the plant growth medium. Hydrogels, known for their efficiency as a water-holding reservoir and nutrient mobilizer in soil, have been utilized in agriculture for five decades [141]. These hydrogels, constructed from superabsorbent polymers, have been widely adopted in the agriculture industry for their roles in soil enhancement, facilitating plant growth in arid conditions, and aiding seed germination [142]. Studies on the application of hydrogels across various soil types and dosages have shown remarkable water absorption properties, absorbing water at a rate 400 times its dry weight and releasing water gradually to reduce herbicide and fertilizer leaching, ultimately improving soil quality, and reducing the need for frequent irrigation [143]. Research findings from a green roof study suggest that a combination of 20% coconut coir, 80% perlite, and a 1.0 kgm^−3^ hydrogel provides optimal plant growth and enhanced ornamental quality, particularly seen in Mentha suaveolens [144]. Additionally, a hydrogel composition of 20% carboxymethyl cellulose (CMC), 20% polyacrylamide (PAM), and oligoalginate sterilized with irradiation at 15 kGy emerges as the most effective plant growth medium compared to coir dust used as a control [145]. These comprehensive investigations highlight the versatility and efficacy of hydrogels in urban farming applications.

### 5.4. Hydrogel as a Potting Medium

The selection of an appropriate potting medium holds a significant role in establishing an environment conducive to optimal plant growth and fostering the healthy development of root systems, both of which are integral aspects of overall plant vitality. While soil has conventionally served as the preferred medium owing to its ubiquitous availability, challenges persist, particularly in handling and transportation, more so in extensive settings such as glasshouses [146]. Soil-based farming practices are vulnerable to soil-borne diseases, with microbial composition acting as a vital determinant of soil health. Hydrogels, characterized by their lightweight nature and rising popularity in agricultural circles, have emerged as a favored potting medium, with a primary emphasis on their capacity for effective water retention to support plant growth. Current research directions are focusing on the exploration of biodegradable hydrogels and their applications in urban farming scenarios, underscoring the importance of water conservation, nutrient retention, and the notable advantages they offer in the cultivation of fruit crops, enhancing sustainability and efficiency in agricultural practices [140,147].

**Figure 11:**
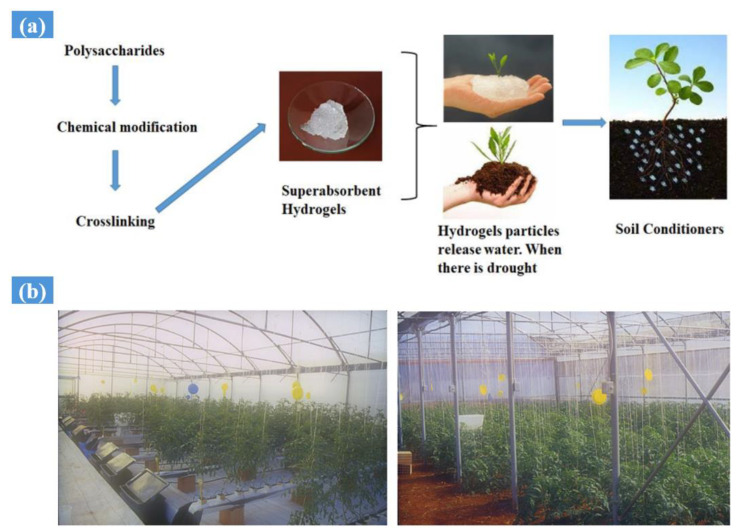
(**a**) Graphical illustration of an agricultural soil conditioner utilizing superabsorbent hydrogels derived from polysaccharides [148]. (**b**) Greenhouse testing locations in Southern Italy for hydrogel experimentation [151].

In a recent study conducted in 2023, a research team examined the potential of hydrogels as an innovative material in agriculture. The study provides a summary of various synthesis methods, types of hydrogels, and crosslinking agents utilized to develop hydrogels tailored for agricultural use (as shown in Figure 11a) [148,149,150]. A research team detailed an experimental tomato cultivation project in Southern Italy, where they introduced a novel humidity sensor incorporating a hydrogel as a sensitive element. This innovative sensor was designed to monitor the moisture levels within the hydrogel in the soil, enabling precise irrigation timing based on real-time moisture conditions (as shown in Figure 11b) [151].

**Figure 12:**
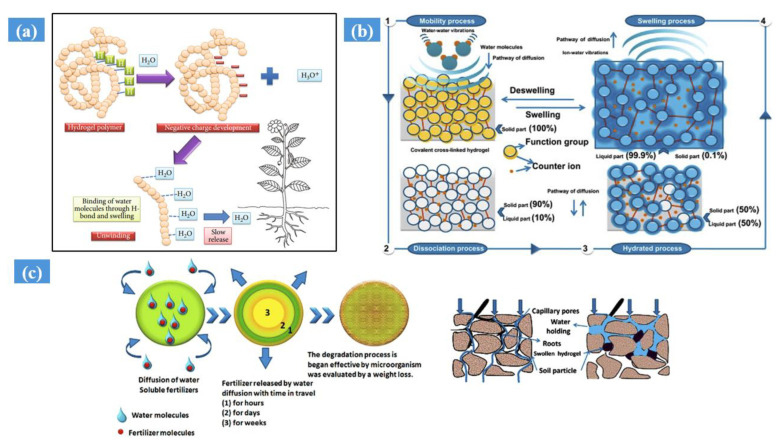
(**a**) Mechanism of hydrogel action in soil applications [152]. (**b**) Swelling mechanism of hydrogels. (**c**) Illustrated procedure of complete fertilizer release from hydrogel and effects of polysaccharide hydrogel on soil texture for plant growth [155].

In 2022, a research group explored the potential of hydrogels in agriculture to boost crop and water productivity in water-scarce environments (as shown in Figure 12a) [152]. This innovative approach aimed to manage water efficiently under water-stressed conditions by preserving soil moisture in the active root zone of crops, thereby minimizing evaporation, deep percolation, and runoff losses. Hydrogels in agriculture, acting as water retention granules, have the unique ability to expand multiple times their original size upon contact with water. By absorbing and retaining significant moisture during periods of heavy rainfall or irrigation, they can subsequently release this stored water back into the soil to meet crop water requirements when the rhizosphere zone undergoes drought conditions [153,154]. This creates negative ions on the polymer chain, leading to chain unwinding and the attraction of water molecules through hydrogen bonding.

In 2023, a research group highlighted the significance of biopolymer-based hydrogels in agriculture and their water-holding capabilities, emphasizing their dual role as soil conditioners and slow-release mechanisms for fertilizers in challenging conditions [155] (as shown in Figure 12b,c). They discussed the use of hydrophilic hydrogels applied during planting or seed coating, primarily serving as carriers for nutrients and enhancers of soil quality [156,157]. Key considerations when selecting a hydrogel for soil enhancement include superabsorbent properties, biodegradability, and chemical crosslinking. By integrating hydrogels with fertilizers in soil, nutrient leaching can be reduced, promoting controlled and gradual nutrient release to improve crop productivity while minimizing fertilizer needs [158]. Studies have shown that fertilizers embedded within hydrogels release nutrients at a slower rate compared to conventional water applications, demonstrating enhanced nutrient efficiency [159,160].

## 6. Conclusions and Future Direction

In conclusion, the synthesis of hydrogels through gamma and electron beam radiation signifies substantial progress in materials science, with pivotal roles spanning across biomedical engineering, wound healing, and agriculture. These sophisticated radiation techniques enable the meticulous control of hydrogel crosslinking, yielding materials with customized properties such as improved mechanical durability, exceptional absorbency, and increased biocompatibility. In the realm of biomedical engineering, these engineered hydrogels excel in applications such as drug delivery systems and tissue engineering scaffolds, offering environments that closely simulate biological tissues. For wound healing applications, radiation-synthesized hydrogels foster an optimal healing environment, speeding up regeneration while facilitating targeted therapeutic delivery. In agriculture, they enhance soil water retention, reduce the frequency of irrigation, and improve the delivery of nutrients, thereby amplifying crop yield even under stringent environmental stresses.

In the future, further improvements could be achieved through the precise optimization of radiation parameters so that these hydrogel systems are more robust in physical and chemical terms and more environmentally friendly. Integrating sophisticated materials like nanomaterials or bioactive agents may spur the development of next-generation hydrogel systems with superior functionalities. Moreover, comprehensive longitudinal in vivo studies to evaluate the long-term safety and effectiveness of these hydrogels, especially within clinical and agricultural frameworks, are imperative. Advances in these areas could markedly expand the practical applications of hydrogels, revolutionizing their use in critical care and sustainable agriculture, particularly in resource-constrained environments. This critical trajectory ensures hydrogels remain at the forefront of technological innovation, meeting the demands of an evolving global market.

## Figures and Tables

**Table 1:** Comparison of gamma, electron beam, and UV radiation for hydrogel synthesis: advantages, disadvantages, applications, and dose ranges.

Synthesis Method	Example	Advantage	Disadvantage	Application in Different Field	Dose Range (KGy)	Ref.
Gama radiation synthesis	Polyethylene glycol diacrylate (PEGDA)	Precise crosslinking	Requires specialized facilities	**Biomedical:** drug delivery systems, tissue engineering	10–25	[54,55]
				**Agriculture:** soil conditioning, water retention		
				**Environmental:** wastewater treatment		
				**Industrial:** smart materials, sensors		
Electron beam irradiation	Polyvinyl alcohol (PVA)	Controlled crosslinking	High equipment costs	**Biomedical:** tissue scaffolds, wound dressings	10–50	[56,57]
				**Food Packaging:** enhanced barrier properties, extended shelf life		
				**Environmental:** pollutant removal, water treatment		
				**Industrial:** tailored materials, coatings		
UV light irradiation	Poly(N-isopropylacrylamide) (PNIPAM)	Cost-effective	Limited depth penetration	**Biomedical:** photo responsive hydrogels, drug release systems	N/A	[58,59]
				**Adhesives:** quick curing		
				**Coatings:** surface modification for bio applications.		

## Data Availability

Not applicable.
